# Influencing factors of glymphatic system during perioperative period

**DOI:** 10.3389/fnins.2024.1428085

**Published:** 2024-09-12

**Authors:** Rui Dong, Wenjie Liu, Yuqiang Han, Zimo Wang, Linhao Jiang, Liwei Wang, Xiaoping Gu

**Affiliations:** ^1^Department of Anesthesiology, Nanjing Drum Tower Hospital, The Affiliated Hospital of Nanjing University Medical School, Nanjing, China; ^2^Department of Anesthesiology, Qingdao Municipal Hospital, Qingdao, China; ^3^Key Laboratory of Anesthesiology and Resuscitation, Ministry of Education, Huazhong University of Science and Technology, Wuhan, China; ^4^Department of Anesthesiology, Xuzhou Central Hospital, Xuzhou, China

**Keywords:** glymphatic system, perioperative, anesthetic drugs, physiology, pathology, AQP4, cerebrospinal fluid

## Abstract

The glymphatic system is a functional cerebrospinal fluid circulatory system that uses peri-arterial space for inflow of cerebrospinal fluid and peri-venous space for efflux of cerebrospinal fluid from brain parenchyma. This brain-wide fluid transport pathway facilitates the exchange between cerebrospinal fluid and interstitial fluid and clears metabolic waste from the metabolically active brain. Multiple lines of work show that the glymphatic system is crucial to normal brain functions, and the dysfunction of the glymphatic system is closely associated with various neurological disorders, including aging, neurodegeneration, and acute brain injury. Currently, it is common to explore the functional and molecular mechanisms of the glymphatic system based on animal models. The function of glymphatic system during perioperative period is affected by many factors such as physiological, pathological, anesthetic and operative methods. To provide a reference for the interpretation of the results of glymphatic system studies during perioperative period, this article comprehensively reviews the physiological and pathological factors that interfere with the function of the glymphatic system during perioperative period, investigates the effects of anesthetic drugs on glymphatic system function and the potential underlying mechanisms, describes operative methods that interfere with the function of the glymphatic system, and potential intervention strategies based on the glymphatic system. Future, these variables should be taken into account as critical covariates in the design of functional studies on the glymphatic system.

## 1 Introduction

**FIGURE 1:**
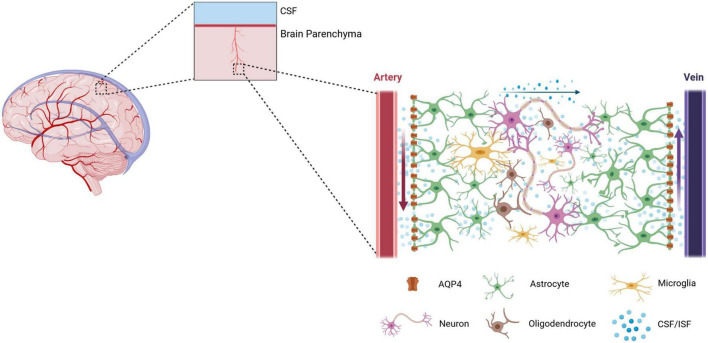
A schematic diagram of the Glymphatic system. The glymphatic system is a brain-wide paravascular pathway that is responsible for clearing interstitial solutes. The glymphatic system consists of four functional processes. Firstly, cerebrospinal fluid (CSF) enters by bulk flow into the periarterial spaces surrounding the arteries that penetrate deep into the brain parenchyma. Here, the perivascular spaces are utilized as “highways” for fast transport of CSF into deep brain regions. Secondly, CSF enters the brain parenchyma from the periarterial spaces. Here, the movement of CSF into the parenchyma is facilitated by the aquaporin-4 (AQP4). Under physiological conditions, AQP4 is mainly expressed on the astrocytic endfeet lining the perivascular space. Thirdly, Cerebrospinal fluid and intertissue fluid (ISF) are exchanged within the brain parenchyma. However, the form of CSF/ISF flow in the brain parenchyma is still controversial. Fourthly, neurotoxic and metabolic wastes from the ISF re-enter the CSF via the perivenous spaces. Eventually, the cerebrospinal fluid containing neurotoxic and metabolic wastes flows through the meningeal lymphatic vessels to the cervical lymph nodes.

Cerebrospinal fluid (CSF) in the subarachnoid space enters the brain parenchyma through the perivascular space (PVS) formed by the cerebral surface arteries and penetrating arteries, exchanges with interstitial fluid (ISF), and leaves the brain parenchyma through the PVS while carrying cellular metabolic wastes and proteins. This functional CSF circulation pathway is called the glial-lymphatic system or the glymphatic system (Iliff et al., 2012), as shown in Figure 1. Aquaporin-4 (AQP4) plays a key role in the glymphatic system. Under normal conditions, AQP4 water channel is expressed primarily in the paravalvular endfeet of astrocytes in a highly polarized manner, where it facilitates the entry of CSF in the PVS into the brain interstitium, which in turn completes the exchange with ISF (Yao et al., 2023). When AQP4 is depolarized, it is highly expressed in the astrocyte cytosol, where it significantly inhibits the entry of CSF into the brain interstitium and impairs the removal of interstitial solutes (Xin et al., 2023).

The adult brain must remove at least 7 grams of waste proteins per day (Chen et al., 2020). In the periphery, metabolic wastes, which includes micromolecule and macromolecules in tissue fluids, can be removed by the lymphatic system. In contrast, the glial-lymphatic system removes toxic proteins and metabolites such as tau and lactate from the brain. In addition, the glial-lymphatic system can deliver biological information and active substances from the choroid plexus and CSF to the brain parenchyma (Gomolka et al., 2023; Harrison et al., 2020; Lundgaard et al., 2017). Therefore, the glymphatic system has a crucial role in maintaining the homeostasis of the brain. The dysfunction of glymphatic system can lead to the accumulation of central toxic metabolites, dysregulation of the brain’s internal environment, neuronal damage, and several related neurological disorders and cognitive impairment (Mestre et al., 2017).

Given its functional importance, the glymphatic system has been a major topic in the neuroscience research field since its discovery in 2012. This article comprehensively reviews the the physiological and pathological factors that interfere with the function of the glymphatic system during perioperative period, investigates the effects of anesthetic drugs and operative methods on the function of glymphatic system and the potential underlying mechanisms, and potential intervention strategies based on the glymphatic system.

## 2 Physiological factors affecting the function of the glymphatic system

**FIGURE 2:**
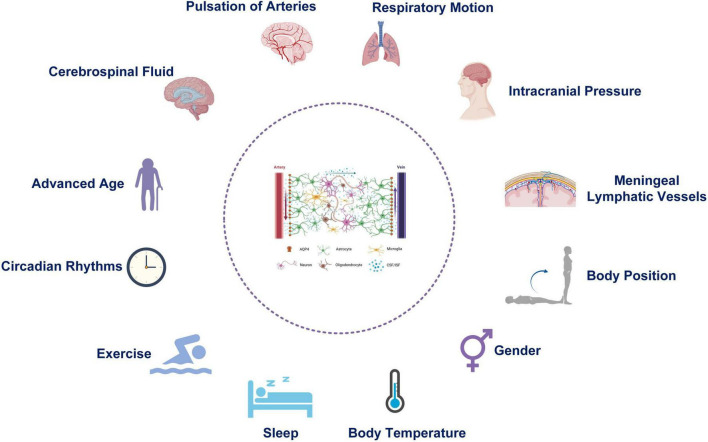
Physiological factors affecting the function of the Glymphatic system.

The function of the glymphatic system can be affected by many physiological factors (Figure 2), which are discussed below.

### 2.1 CSF produced by the choroid plexus

CSF is principally responsible for the physiological functions of the glymphatic system, as a crucial macroscopic driver. CSF fills the brain and the spinal cord and is involved in maintaining the balance of the central internal environment, providing nutrition, and regulating intracranial pressure (Czarniak et al., 2023; Ghersi-Egea et al., 2018). The choroid plexus continuously produces CSF, whose total amount remains relatively constant (Ghersi-Egea et al., 2018). From the subarachnoid space, CSF is driven into the PVS by a combination of arterial pulsatility, respiration, slow vasomotion, and CSF pressure gradients. AQP4 then transports the subsequent of CSF into the dense and complex brain parenchyma (Iliff and Nedergaard, 2013; Iliff et al., 2012). CSF movement into the parenchyma drives convective ISF fluxes within the tissue toward the perivenous spaces facilitates CSF-ISF exchange and metabolite clearance within the glymphatic system (Jessen et al., 2015). Both inflammatory stimulation of the brain and an altered brain microenvironment affect the rate of CSF production (Kaur et al., 2016), and physically block perivascular flow and influx of CSF (Ivan et al., 2020; Rustenhoven et al., 2021). A recent study confirmed the glymphatic flow is impaired in response to stimulation by peripheral inflammation (Manouchehrian et al., 2021). It seems self-evident that the damaged glymphatic system further accelerates the accumulation of inflammatory cytokines and metabolic wastes, which create a vicious cycle to perpetuate neuroinflammation. Thus, the choroid plexus may enhance the clearance of the glymphatic system by modulating CSF production in response to changes in the internal environment (Johnson et al., 2020).

### 2.2 Pulsation of cerebral surface arteries and penetrating arteries

CSF enters the brain parenchyma through PVS around arteries, mixes with ISF to absorb waste, and leaves the brain through PVSs around veins, during which arterial pulsation acts as a vascular pump (Mestre et al., 2018). Therefore, arterial pulsatility is an important driving force of CSF flow. Administration of the adrenergic receptor agonist dobutamine to mice enhances arterial pulsation, increasing entry of CSF into the brain parenchyma. In contrast, ligation of the right internal carotid artery results in a 50% decrease in arterial pulsation, and injection of a tracer 0.5 h later has revealed a decreased rate of exchange of CSF with ISF (Iliff and Nedergaard, 2013). Notably, the pulsatility of the penetrating parenchymal arteries, which play a major role in the glymphatic system, is higher than that of the surface arteries. Bedussi et al. found that the flow of CSF in the periarterial space has a pulsatility consistent with the heart rhythm. Although this pulsatility itself does not increase the inflow of CSF into the periarterial gap, it promotes the entry of CSF into the brain parenchyma and enhances exchange with ISF (Bedussi et al., 2018). Harrison et al. through diffusion tensor magnetic resonance studies, have found that the pseudo-diffusion coefficient of PVS increases 300% during cardiac systole compared with cardiac rest (Harrison et al., 2018), suggesting that arterial pulsation enhances the diffusion movement of the CSF in PVS. Anatomically, the periarterial gap is larger than the perivenous gap (Bedussi et al., 2018), thereby creating a low resistance channel for CSF to flow into the brain parenchyma, and explaining why CSF enters the brain parenchyma along the periarterial gap, rather than the venous gap.

Given that arterial pulsation is affected by blood pressure and heart rate, changes in blood pressure and heart rate can also affect the function of the glymphatic system. The effect of heart rate on the glymphatic system remains controversial. The inflow of CSF in the glymphatic system is inversely proportional to the heart rate (Hablitz et al., 2019). However, Kyrtsos et al. have studied the glymphatic system through mathematical modeling and found that for every 10 beats/min decreases in heart rate, the brain parenchyma accumulates nearly 20% additional β-amyloid (Aβ). In contrast, a 30 increase in heart rate decreases Aβ levels in the brain parenchyma by nearly 30% (Kyrtsos and Baras, 2015). Multiple independent studies have found that hypertension inhibits vascular pump movement, affects ordinary CSF hydrodynamics, and suppresses the substance clearance function of the glymphatic system (Mestre et al., 2018; Mortensen et al., 2019; Zhang et al., 2021; Zhou et al., 2020), explaining why hypertension causes accumulation of Aβ in the brain (Drews et al., 2019).

### 2.3 Respiratory movements

In contrast to the positive pressure-driven effect of arterial pulsation on the entry of CSF into the cerebral parenchyma, respiratory movements mainly affect venous contraction and have a “suction” effect on the ISF. Respiratory movements cause venous distension and collapse, thereby resulting in periodic narrowing and widening of the perivenous space, which in turn facilitates the flow of ISF out of the cerebral parenchyma (Dreha-Kulaczewski et al., 2017). Using magnetic resonance electroencephalography, Kiviniemi et al. demonstrated that respiratory movements drive CSF flow in the glymphatic system by affecting the perivenous space. Respiratory movements affect the direction and speed of CSF flow. The rate of CSF flow increases in the inspiratory phase, and holding the breath significantly inhibits CSF flow (Dreha-Kulaczewski et al., 2015). Therefore, in patients with obstructive sleep apnea, compared with unaffected individuals, the increased levels of Aβ and neurogenic proteins in the CSF may be due to the abnormal function of the glymphatic system caused by apnea, thereby resulting in the deposition of Aβ and neurogenic proteins in the brain (Ju et al., 2016).

### 2.4 Body position

Using sleep dysfunction rats as study subjects, Lee et al. found that right lateral decubitus results in rapid clearance of Aβ from the brain (Lee et al., 2015). Preliminary multicenter research has also indicated that a supine sleep position is independently associated with neurodegenerative diseases and that a lateral sleep position promotes neurotoxic protein clearance and prevents the development of neurodegenerative diseases to some extent (Levendowski et al., 2019). The specific mechanisms through which postural changes affect the function of the glymphatic system are unknown. However, postural changes can trigger a range of physiological changes in the cardiovascular system, respiratory system, and intracranial pressure (Kim et al., 2011), all of which may affect the function of the glymphatic system. Therefore, this interfering factor should not be ignored, and researchers should be aware of the interference of postural factors when assessing the function of the glymphatic system through various imaging techniques to standardize the results.

### 2.5 Sleep

The need for sleep in all species reflects the need for the brain to enter a state in which it can eliminate neurometabolic waste (Jessen et al., 2015). The waste removal function of the glymphatic system meets many of the criteria for “sleep needed” proposed by Borbely (Borbély, 1982). In mice, the periarterial to interstitial: the overall CSF flow are higher in the sleeping state than in the awake state, and the rate of Aβ clearance is more than twice that in the awake state (Xie et al., 2013). Clinical studies also indicated that the glymphatic system operates mainly in the sleeping state (Demiral et al., 2019). The mechanisms through which sleep enhances the glymphatic system are as follows: 1) during sleep, the body’s adrenergic activity decreases, and the extracellular space in the brain enlarges, reducing the resistance to CSF flow in the brain parenchyma and increasing the efficiency of glymphatic clearance (Xie et al., 2013). 2) During sleep, the volume of CSF increases, and the driving force is enhanced (Demiral et al., 2019). 3) During sleep, the polarization of AQP4 on astrocyte paravalvular end-feet is enhanced, thereby promoting the entry of CSF from the periarterial space into the brain parenchyma, where it participates in metabolic waste removal (Hablitz et al., 2020). The above studies have demonstrated that sleep has an essential role in the clearance function of the glymphatic system and its maintenance of metabolic homeostasis, and may influence the pathophysiological processes in the development of nervous system diseases. Satio et al. found that the index of diffusivity along the perivascular space (ALPS index) showed significant negative correlations with the Pittsburgh Sleep Quality Index (PSQI) scores of all the components, suggesting that glymphatic system impairment contributes to sleep disruption in young adults (Saito et al., 2023). Winer et al. have observed a faster rate of intracerebral Aβ accumulation in people with low sleep efficiency (total sleep as a percentage of total bedtime) (Winer et al., 2020). A large sample long-term cohort study has found that a sleep duration shorter than 6 h is associated with a 30% increased risk of AD in middle-aged and older participants (Sabia et al., 2021). A recent clinical study has shown that chronic insomnia in middle-aged and elderly people is associated with glymphatic system dysfunction and cognitive decline (Jin et al., 2024). Given that the glymphatic system has a scavenging effect on Aβ protein in the brain, in-depth exploration of the effects of sleep on the scavenging function of the glymphatic system should open new horizons for revealing the mechanism of sleep and the diagnosis and treatment of brain diseases associated with it (Miao et al., 2024).

### 2.6 Circadian rhythms

The function of the glymphatic system is enhanced during sleep and inhibited in the waking state, showing a distinct circadian rhythm (Cai et al., 2020). Studies have revealed that this rhythmic variation is controlled by the biological clock (Vizcarra et al., 2024). Levels of tau protein and lactate in the hippocampal ISF correlate with the sleep-wake cycle, with neuronal activity producing the highest levels of tau and lactate during the dark phase when mice are active, and the lowest levels during the light phase, when mice are resting or sleeping (Holth et al., 2019). These findings are consistent with the rhythm of activity of the glymphatic system; in addition, the glymphatic system is involved in the clearance of lactate and tau proteins from the brain parenchyma (Cao et al., 2018). The rhythmicity of the glymphatic system manifests in three aspects. First, the circadian rhythms of AQP4 polarization are controlled by the clock gene BMAL1 (Lananna et al., 2018). Second, the choroid plexus, which generates CSF, exhibits robust circadian behavior and influences the suprachiasmatic nucleus, which is the biological rhythm control center (Myung et al., 2018). Third, circadian rhythm differences are observed in the distribution rate of CSF in different brain regions (Hablitz et al., 2020).

### 2.7 Gender

The effect of gender on the function of the glymphatic system is debatable. In 2017, researchers proposed Diffusion Tensor Image Analysis Along the Perivascular Space (DTI-ALPS), a non-invasive method that reflects glymphatic function, and the ALPS index, which indirectly reflects the speed of fluid motion in PVS (Taoka et al., 2017). A clinical study found that females have a significantly higher ALPS index compared to males, suggesting females have stronger glymphatic transport (Zhang et al., 2021). In contrast, in a study on C57BL/6 mice, Giannetto et al. found no differences in CSF influx or subregion-dependent tracer distribution at different ages between sexes, however, female mice show stronger circadian rhythmicity than male mice (Giannetto et al., 2020).

### 2.8 Body temperature

Anesthesia decreases the temperature of the brain by approximately 3–4°C (Wang et al., 2014). According to Brownian motion theory, hypothermia is assumed to decrease the diffusion of water molecules in the brain parenchyma and to inhibit the function of the glymphatic system. Gu et al. found that a considerable reduction in glymphatic drainage function following TBI, which was aggravated by further hypothermia (Gu et al., 2023). However, numerous studies have confirmed the neuroprotective effect of hypothermia (Duan et al., 2020). Therefore, this relationship and neuroprotective effects must be investigated to better understand the mechanisms underlying hypothermia’s neuroprotective effects.

### 2.9 Advanced age

The function of the glymphatic system decreases significantly with age (Giannetto et al., 2020). Han et al. found that glymphatic efflux activity assessed via DTI-ALPS decreased with age over in a large age range of 21–75 years (Han et al., 2023). The aging-induced decrease in the function of the glymphatic system is associated with the depolarization of AQP4 (Kress et al., 2014) as well as impaired CSF production by the choroid plexus during aging, progressive reduction and degeneration of the arachnoid villi (Johanson et al., 2008), and weakened arterial pulsation due to arterial wall sclerosis (Kress et al., 2014). Impaired integrity of the meningeal lymphatic vessels (Ahn et al., 2019) is also responsible for the decline in the function of the glymphatic system with advanced age. Therefore, glymphatic system dysfunction is an essential feature of brain aging.

### 2.10 Plasma osmolality and intracranial pressure

Elevation of plasma osmolality in mice by intraperitoneal injection of hypertonic saline does not significantly increase blood-brain barrier permeability; in contrast, the brain shows 125% increased uptake of a fluorescent tracer injected via the large occipital pool and 70% increased inflow (Plog et al., 2018). Even in awake mice, plasma hyperosmolarity increases tracer influx into the PVS and enhances glymphatic system function (Plog et al., 2018). Plasma hyperosmolarity may be associated with increased ISF outflow and decreased intracranial pressure caused by hyperosmotic fluid. Bedussi et al. found that the PVS, subarachnoid space, and brain pool are not independent, instead, CSF in the aforementioned spaces is connected, and changes in pressure in the subarachnoid space and brain pool also affect the exchange of substances in the PVS (Bedussi et al., 2017).

### 2.11 Meningeal lymphatic vessels

In 2015, Kipnis’ team discovered meningeal lymphatic vessels (mLVs) in the superior sagittal sinus, sinus sink, and dorsal transverse sinus (Louveau et al., 2015). This anatomical discovery was a breakthrough in brain science research in recent decades that overturned the previous notion that lymphatic vessels do not exist in the brain. In 2019, Ahn (Ahn et al., 2019) discovered basal mLVs located at the base of the skull and described their specific morphological features (Table 1). Basal mLVs are more conducive to the collection and drainage of CSF than dorsal mLVs. Traditionally, CSF was believed to be absorbed by arachnoid granulations into the venous blood, but the discovery of mLVs has provided objective conditions for the clearance of CSF from the perivenous space in the glymphatic system (Ma et al., 2017). Da Mesquita has shown that disruption of mLVs causes dysfunction of the glymphatic system without increasing intracranial pressure and leads to cognitive dysfunction in mice (Da Mesquita et al., 2018). Under invasive stimulation with implanted brain electrodes, mLVs appear to proliferate responsively, and proliferating mLVs enhance the function of the glymphatic system (Hauglund et al., 2020). Induction of mLVs production with recombinant human vascular endothelial growth factor enhances the clearance of Aβ in the brain parenchyma (Wen et al., 2018). However, notably, the integrity of the mLVs and CSF drainage regresses with age and may be a factor in the decline in function of the glymphatic system in advanced age (Chen et al., 2024).

**TABLE 1:** Differences between dorsal mLVs and basal mLVs.

	Dorsal mLVs	Basal mLVs
Anatomical position	Traveling along with the sagittal and transverse sinuses	Proximity to the subarachnoid space, traveling along with the petrosal and sigmoid sinuses
Tube lumen diameter	Small	Big
Intraluminal valve structure	None	Exist
Lymphatic endothelial cell junction mode	Immature state, continuous closed zipper-like connection	Similar structure to peripheral lymphatic vessels, mainly connected in a discontinuous closed button-like manner
Branches	Most have no branch	Abundant capillary branches with bluntly rounded ends
Drainage of large molecules in the cerebrospinal fluid	Weak	Strong
Sensitivity Analysis	Strong	Weak
Where to divert traffic	Deep cervical lymph nodes and superficial cervical lymph nodes	

### 2.12 Level of voluntary exercise

The independent studies have found that the level of voluntary exercise significantly enhances the function of the glymphatic system and improves cognition in mice (He et al., 2017). Furthermore the increased functional activity of the glymphatic system is attributable to physiological adaptation by prolonged exercise (5 weeks) rather than being a temporary effect of exercise (von Holstein-Rathlou et al., 2018). Exercise inhibits reactive astrocyte proliferation and reverses the depolarization of AQP4 in the brains of aged mice (14–16 months of age) (He et al., 2017). In contrast, in young mice (9 weeks of age), exercise does not affect the astrocyte state or the polarization state of AQP4 (von Holstein-Rathlou et al., 2018), suggesting that the mechanism through which autonomous exercise enhances the function of the glymphatic system varies with age.

## 3 Pathological factors affecting the function of the glymphatic system

### 3.1 Neuroinflammation

Neuroinflammation can affect the function of the glymphatic system, which is mainly reflected in: (1) Neuroinflammation triggers astrocyte activation, changes the morphology of astrocytes, and influences the CSF flow pattern within the glymphatic system. Meanwhile, the activation of astrocytes can also release inflammatory mediators, further aggravating neuroinflammation. (2) Neuroinflammation triggers depolarized AQP4 expression and glymphatic system dysfunction (Liu et al., 2020). (3) Neuroinflammation attracts neuroimmune cells and inflammatory factors, which then accumulate in the PVS and directly affects the inflow and outflow of CSF (Rasmussen et al., 2022). (4) Neuroinflammation affects the distribution of CSF in the PVS. Erickson et al. researched that systemic LPS-exposure reduced bulk flow of CSF, and decreased amyloid-β clearance (Erickson et al., 2012). Manouchehrian et al. injected LPS (1mg/kg) into the abdominal cavity of mice, and the results showed that compared with the control group, the activation markers of microglia in LPS-mice increased, and the perivascular CSF flow decreased significantly (Manouchehrian et al., 2021). However, the polarization degree of AQP4, astrocyte markers and cerebral blood flow were not significantly changed (Manouchehrian et al., 2021).

### 3.2 Alzheimer’s disease (AD)

The glymphatic system is involved in the clearance process of various pathological proteins, and 40% to 80% of large protein molecules and solutes are cleared through this pathway. The deposition of abnormal proteins (Aβ, tau,α synuclein)in the brain is an important pathological feature of the occurrence and development of neurodegenerative diseases, and the elimination of these core pathogenic substances is the key to prevent or delay the progression of the disease (Harrison et al., 2020; McKnight et al., 2021). Relative to the young, clearance of intraparenchymally injected Aβ was impaired by 40% in the old mice (Kress et al., 2014). The polar distribution of AQP4 in the paravascular channel in the brain of aged mice was lost: AQP4 was no longer confined to the astrocyte terminal, but was more evenly distributed on the entire astrocyte cell membrane. Aging contributes to the dysfunction of the glymphatic system, resulting in the accumulation of Aβ and ultimately developing AD (Kress et al., 2014). The discovery of the glymphatic system complements the transport pathway of Aβ, explaining the question of the limited transport capacity of carrier proteins on BBB and the excessive distance of some brain cells, and so on (Cui et al., 2021). Meanwhile, the glymphatic system can produce convection when it transports substances, and this flow can increase the scope of BBB domination and better remove metabolic wastes such as Aβ.

### 3.3 Parkinson’s disease (PD)

PD is the second most prevalent neurodegenerative condition in the world, characterized by the degeneration and loss of dopaminergic neurons (Huang et al., 2023). The symptoms of PD are a combination of motor and non-motor symptoms. Motor symptoms involve static tremors, rigidity, and bradykinesia, while non-motor symptoms include sleep disorders, constipation, and hyposmia (Keir and Breen, 2020). Furthermore, A biomarker of PD, α-synuclein (α-syn), is thought to be involved in the pathogenesis of PD when it is abnormally folded. When abnormally folded α-syn proteins accumulate in the brain, they form Lewy bodies, triggering a series of clinical symptoms (Abdelmoaty et al., 2023). Decreased AQP4 expression leads to further accumulation of α-syn in the CNS and exacerbates progressive pathology and motor aberrations (Cui et al., 2021). Glymphatic influx of CSF tracer was reduced in PD mice, accompanied with perivascular aggregation of α-synuclein and impaired polarization of aquaporin 4 expression in substantia nigra. Cervical lymphatic ligation aggravated glymphatic dysfunction of PD mice, causing more severe accumulation of α-synuclein, glial activation, inflammation, dopaminergic neuronal loss and motor deficits. In addition, AQP4 deficiency decreases the clearance rate of α-syn in the brain parenchyma, indicating that the reduced clearance rate of α-syn is closely related to the dysfunction of the glymphatic system, which may be a contributing factor to the occurrence of PD (Zou et al., 2019). Erin K et al. found that global and regional perivascular space volumes significantly increased in 470 PD patient’s MRI images, indicating a difference in the volume of PVS spaces between patients and healthy individuals in the medial orbitofrontal region and the banks of the superior temporal regions (Donahue et al., 2021). What’s more, an earlier study with 271 individuals revealed that basal ganglia PVS enlargement is linked to cognitive impairment in PD patients, suggesting that it could be a valuable clinical measure (Park et al., 2019).

### 3.4 Traumatic brain injury (TBI)

TBI is a risk factor for neurodegeneration such as dementia and AD (Moretti et al., 2012). The overall expression of AQP4 in cortex and striatum increased in mice after TBI, but the distribution of AQP4 around blood vessels decreased, and the polarity distribution of AQP4 in most mice did not recover after 4 weeks (Lundgaard et al., 2017; Ren et al., 2013). Meanwhile, the function of transport and clearance of tau protein in mouse glymphatic system remained impaired within 4 weeks after TBI, and the phosphorylated tau protein in brain showed a widespread increase after 4 weeks (Lundgaard et al., 2017). Dysregulation of the hypothalamus, pineal gland and brain stem after TBI, which further leads to changes in sleep structure and disturbances in sleep regulation. This sleep disturbance will lead to impaired glymphatic function and decreased clearance, resulting in a large amount of Aβ and Tau protein deposition (Xie et al., 2013). Therefore, dysfunction of the glymphatic system leads to reduced protein clearance and abnormal protein aggregation, which may be the mechanisms of secondary degeneration after TBI. Early intervention of the glymphatic system can be considered to maintain its normal function of protein elimination and prevent dementia following TBI.

### 3.5 Diabetes

The brain is a primary organ that targets insulin, and disrupting insulin metabolism compromises the function of neurons and glial cells during diabetes mellitus (DM) (Arnold et al., 2018; Tumminia et al., 2018). Moreover, a large amount of evidence indicates that diabetes-induced cognitive impairment is closely related to glymphatic system dysfunction. Jiang et al. investigated the effect of diabetes on the glymphatic system and the link between alteration of glymphatic clearance and cognitive impairment in Type-2 diabetes mellitus rats (Jiang et al., 2017). The results revealed that clearance of CSF contrast agent Gd-DTPA from the interstitial space was slowed by a factor of three in the hippocampus of Type-2 diabetes mellitus rats compared to the non-DM rats. Cognitive deficits detected by behavioral tests were highly and inversely correlated to the clearance of Gd-DTPA contrast and fluorescent tracer in the hippocampus of Type-2 diabetes mellitus rats. Type-2 diabetes mellitus suppresses clearance of ISF in the hippocampus and hypothalamus, resulting in Type-2 diabetes mellitus-induced cognitive deficits (Jiang et al., 2017). MRI showed that lower CSF diffusivity along the PVS, the CSF bulk speed in the para-vasculature network is low, and the clearance rate of the brain parenchyma decreased in a typical DM brain (Davoodi-Bojd et al., 2019; Yang et al., 2020). Notably, increasing evidence has shown that DM affects astrocyte activation and AQP4 expression and polarization distribution (Abdul et al., 2021). The expression of AQP4 in the hippocampus of diabetic rats was significantly reduced (Zanotto et al., 2017). With the continuous increase of blood glucose, Fukuda et al. observed the change of perivascular AQPs from AQP4 to AQP1 was observed in rats with spontaneous diabetes (Fukuda et al., 2010). Diabetes compromises the integrity of the conceptual neurovascular unit in the hippocampus, leading to increased blood-brain barrier (BBB) permeability, vascular remodeling, higher levels of hippocampal cell mortality, greater astrocyte reactivity, disruption of the AQP4 polarity within the astrocytes, and ultimately impaired cognitive function (Ward et al., 2019; Zhang et al., 2019). Furthermore, MRI can give sensitive quantitative signs of glymphatic dysfunction along the evolution of DM, making it a promising tool for the early identification of DM with clinical applicability (Boyd et al., 2024).

### 3.6 Hypertension

Chronic hypertension leads to stiffness and reduced elasticity of blood vessel walls, which may reduce the effectiveness of arterial pulsation as a driver of CSF-ISF, thereby impacting glymphatic transport. Mortensen et al. showed that the ventricular system anatomy and associated CSF transport pattern differed dramatically between spontaneously hypertensive (SHR) and normotensive Wistar-Kyoto rats (WKY): Young and adult SHR rats displayed a 5–6-fold larger lateral ventricle volume compared to WKY rats, along with enlargement of cisterna magna (CM) volume and about 10% loss of brain volume (Mortensen et al., 2019). They applied a one-tissue compartment model to estimate the influx rate of Gd-DOTA from CSF to the brain as well as the efflux rate from the brain. Kinetic analysis demonstrated decreases in both glymphatic influx and efflux, glymphatic transport is impaired. Reduced glymphatic influx has previously been associated with impaired glymphatic system function, and thus a reduced ability of the brain to be cleared of macromolecules and fluids. Arterial hypertension induces a change in vessel dynamics that reduces perivascular pumping, decreases the net flow of CSF in PVSs, which will decrease the clearance of brain waste (Mestre et al., 2018).

## 4 Effects of different operations on the glymphatic system and their mechanisms

### 4.1 Operative type

Cervical lymph node is an important downstream structure through which CSF flows out of the skull through mLVs, and deep cervical lymph nodes (dcLNs) and submandibular lymph nodes are the main excretion sites of solute and metabolic waste in glymphatic system (Louveau et al., 2015). Meningeal lymphatic vasculatures are connected to dcLNs, and drain macromolecules and immune cells from the CSF into the peripheral circulation (Wang et al., 2019). Xue et al. found that glymphatic transport and drainage to submandibular and deep cervical lymph nodes were reduced under ketamine/xylazine (KX) anesthesia. Furthermore, glymphatic transport was increased in dcLNs in mice anesthetized with KX compared to ISO (Xue et al., 2020). Ligation of dcLNs blocks glymphatic system drainage, aggravates brain Aβ and Tau accumulation, resulting in neuroinflammation, loss of synaptic proteins, impaired AQP4 polarization, and deficits in cognitive and exploratory behaviors (Cao et al., 2018). When extracranial lymphatic drainage is dysfunctional, mislocalization or disruption of AQP4 expression may promote Aβ-related pathophysiology. Increased intracranial pressure after traumatic brain injury can lead to meningeal lymphatic dysfunction, which makes the brain more prone to severe neuroinflammation and cognitive deficits (Bolte et al., 2020). Moreover, Transverse sinus stenosis (TSS) is a common finding in idiopathic intracranial hypertension (IIH). There was a significant association between the increasing extent of TSS, declining glymphatic clearance, and lower glymphatic flow (Schartz et al., 2024). This suggests that we should also pay attention to the functional changes of the glymphatic system in patients with craniocerebral operation

### 4.2 Operative position

The transport and clearance efficiency of the glymphatic system was the highest in lateral position, the second in supine position, and the lowest in prone position (Ren et al., 2021). However, most patients are in the supine position for a long time during perioperative period, so posture or gravity may also have a regulatory effect on the glymphatic system. A MRI experiment showed that the right lying position is the most favorable position for lymphatic transport, which is consistent with the most preferred sleeping position of humans (Benveniste et al., 2019). In addition, both humans and animals changes their positions several times during a normal sleep cycle, and these rapid postural changes may also affect the function of the glymphatic system (Benveniste et al., 2019). Although the mechanism of the effect of body position on the function of the glymphatic system is not fully understood, it can be considered as an explanation through affecting the cardiovascular system and respiratory patterns based on the current research.

## 5 Effects of different anesthetics on the glymphatic system and their mechanisms

The most severe adverse effects of anesthetic drugs are inhibition of the respiratory system, circulatory system, and thermoregulatory system. Theoretically, all anesthetic drugs that can alter the above physiological factors can affect the function of the glymphatic system. However, controversies regarding the influences of different anesthetic drugs on the glymphatic system have been observed in recent years. The anesthetic drugs currently used in the study of the glymphatic system in living animals and their pharmacological characteristics are shown in Table 2.

**TABLE 2:** Anesthetic drugs commonly used in glymphatic system.

Drug name	Pharmacological effects	Advantages	Disadvantages	Applied dose in mice	Applied dose in rats
Isoflurane	The positive variant modulator of chloride channels of GABA receptor; NMDA receptor, K2P channel and HCN channel antagonist	1. Inhaled anesthetics, not metabolized by the liver and kidneys, rapid induce and recovery 2. mild cardiovascular depression 3. Good muscle relaxation effect	1. Reduce peripheral arterial resistance 2. Moderate respiratory depression	Induction: 2–4% Maintenance: 1–3%	Induction: 1.5–4% Maintenance: 0.4–2.5%
Ketamine	Antagonism of NMDA receptors	1. “Isolated anesthesia” without suppression of the central nervous system 2. No depression of respiration and cardiac output 3. Strong analgesic effect	1. Decrease heart rate and blood pressure and affect brain oxygenation 2. Muscle stiffness 3. hepatic and renal toxicity 4. Recovery hyperresponsiveness and ataxia	80–120mg/kg (i.p.)	80–120mg/kg (i.p.)
Methylphenothiazine Dexmedetomidine	Agonism of α2 adrenergic receptors	1. Sedative-hypnotic effect 2. Large safety range, no accumulation	1. Combined with ketamine, lowering blood pressure significantly 2. May cause bradycardia and respiratory depression	1. Methylphenothiazine 10–20 mg/kg (i.p.) 2. dexmedetomidine; initial dose 0.015 mg/kg (i.p.), maintenance dose 0.015–0.020 mg/kg/h (s.c.), or single one-time infusion 0.2 mg/kg	1. Methylphenothiazine 10–20mg/kg (i.p.) 2. dexmedetomidine; initial dose 0.015 mg/kg (i.p.), maintenance dose 0.015–0.020 mg/kg/h (s.c.), or single one-time infusion 0.5 mg/kg
Phenobarbital Pentobarbital	Enhancement of GABA-mediated Cl endocytosis	1. Sedative and hypnotic effects 2. Better muscle relaxation effect	1. Inhibits sympathetic nerves and lowers blood pressure 2. Poor analgesic-muscarinic effect of conventional dosing 3. Dependent on liver metabolism 4. Poor analgesic effect 5. Respiratory depression	Pentobarbital 60mg/kg (i.p), 50mg/kg (i. v.)	Pentobarbital 5–10mg/kg (i.v.) Phenobarbital 40mg/kg (i.p.)

### 5.1 Ketamine and xylazine

Ketamine and xylazine (KX) were the first anesthetic drugs used in glymphatic system research (Iliff et al., 2012). The effects of KX on the glymphatic system are currently controversial. Groothuis found that solute removal from extracellular space (ECS) of the brain is 100 times slower in pentobarbital-anesthetized rats than in KX-anesthetized rats (Groothuis et al., 2007). Hablitz et al. suggested that KX improves the function of the glymphatic system by enhancing slow-wave oscillations and decreasing the heart rate (Hablitz et al., 2019). However, von Holstein-Rathlou et al. found that KX anesthesia in mice, compared with the awake state, does not enhance the function of the glymphatic system (von Holstein-Rathlou et al., 2018). A recent study using high-resolution 3D FISP-MRI found that CSF flux and flow rate are significantly higher in mice under KX anesthesia than isoflurane anesthesia. Moreover, the CSF contrast agent diffuses along the Willis loop in mice under KX anesthesia, with a portion diffusing toward the brain parenchyma along with the periarterial space and another portion flowing in large volumes along with the PVS and through the nasal turbinates and pharynx lymphatic vessels. In the isoflurane anesthesia state, most CSF does not enter the brain parenchyma but flows towards the spinal cord and cranial ganglia (Stanton et al., 2021).

### 5.2 Dexmedetomidine

Dexmedetomidine is a selective α2 adrenergic receptor agonist that hyperpolarizes locus ceruleus neurons, decreases norepinephrine release, and exerts hypnotic effects (Keating et al., 2012). By injecting α and β receptor blockers into the CSF, Xie found an increase in ISF volume and enhanced transport clearance of the glymphatic system, and a close correlation with cortical slow-wave activity (Xie et al., 2013). Benveniste et al. found that, compared with isoflurane anesthesia alone, dexmedetomidine combined with low-dose isoflurane anesthesia increases the CSF volume in rats by 2% and the glymphatic system transport efficiency by 32%—effects that may be associated with dexmedetomidine inhibition of the function of the locus ceruleus noradrenalin system (Benveniste et al., 2017). Dexmedetomidine improves the circulatory capacity of the glymphatic system in young mice following repeated exposure to sevoflurane and enhances their long-term learning and working memory abilities (Wang et al., 2024). Studies have confirmed that inhibition of the activity of the locus ceruleus noradrenaline system improves the function of the glymphatic system (DiNuzzo and Nedergaard, 2017). On this basis, we hypothesize that anesthetic drugs with noradrenergic inhibitory function might increase the function of the glymphatic system. Many previous studies have confirmed that dexmedetomidine prevents the development of postoperative cognitive dysfunction, and the glymphatic system provides a new mechanism for its cerebral protective effect: improving postoperative cognitive function by improving the clearance of metabolites from the brain. Encouragingly, dexmedetomidine has been shown to promote the distribution of intrathecally injected drugs in the brain by accelerating the lymphocyte circulation (Lilius et al., 2019), suggesting that anesthetic drugs can enhance the function of the glymphatic system, facilitate drug delivery to the brain, and greatly enrich the content of anesthetic therapeutics.

### 5.3 Isoflurane

Isoflurane is currently the most commonly used inhaled anesthetic drug in animal experiments. It is mainly eliminated by the lungs; moreover, it induces rapid recovery, does not inhibit cardiac function, and can reduce peripheral circulatory resistance. General anesthesia with isoflurane inhibits the function of the glymphatic system (Gakuba et al., 2018). The effects are dose-dependent, causing a decrease in CSF drainage by the glymphatic system and increasing drainage through the cerebral nerve and sieve plate channels (Gakuba et al., 2018). Isoflurane anesthesia facilitates glymphatic system function: 4% isoflurane anesthesia (1–6 min), compared with no anesthesia, increases the clearance efficiency of the glymphatic system in rats (Taoka et al., 2018). However, one study has found that long-term isoflurane anesthesia (4.2% isoflurane for induction and 1.5% isoflurane in 30% oxygen at a flow rate of 2–3 L/min for maintenance for 6 h) causes blunted glymphatic system by inducing AQP4 depolarization, enhanced the AQP4 polarization can alleviate the glymphatic system malfunction and reduce the neuroinflammatory response (Dong et al., 2024).

### 5.4 Anesthesia state and the glymphatic system

Besides the influence of anesthetic drugs on the function of the glymphatic system by affecting various body physiological parameters, norepinephrine system activity in nucleus ceruleus and brain waves, some studies have suggested that the anesthetic state itself also affects the function of the glymphatic system. The outflow pathway of CSF in the brain is divided into two major parts: flow to the periphery and the glymphatic system. Ma et al. found that in the anesthetized state (ketamine +medetomidine), CSF flows mainly to the periarterial space, whereas the rate of transfer to the periphery (dCLNs and mandibular lymph nodes) slows. In contrast, in the awake state, CSF flows mainly to the periphery (Ma et al., 2019). However, the validity of this finding under the effects of other anesthetic drugs must be further explored. Preliminary studies have indicated that the depth of anesthesia also affects the function of the glymphatic system (Hauglund et al., 2020), but this possibility must be further verified in rigorously designed experiments.

## 6 Potential intervention strategies based on the glymphatic system

As a highly selective physical barrier, the BBB is an important structure for maintaining brain homeostasis, which can prevent pathogens and toxic substances from entering the central nervous system. However, this structure also prevents many therapeutic drugs from entering the brain, making the treatment of brain diseases difficult. Therefore, a safe and efficient new brain drug delivery strategy has become an important research target for the treatment of brain diseases. Zhao et al. used indocyanine green (ICG)-loaded PLGA nanoparticles as a drug delivery model, subcutaneous injection at neck near local lymph node, and then particles accumulate to dCLNs and continuously diffuse into lymphatic vessels and CSF via the transportation of immune cells, therefore leading to effective brain delivery bypassing BBB (Zhao et al., 2020). Shi et al. exploit a nanostructure, Nano-plumber, that allowing for a sustainable and orderly regulation of the microenvironment to promote long-term neurological recovery. Nano-plumber reverses the injury microenvironment by suppressing microglia and astrocytes activation and promoting the drainage of meningeal lymphatic vessels, and significantly improves the neurological function of rodents with TBI (Tong et al., 2023).

Low-intensity noninvasive transcranial ultrasound may be used to increase the whole-brain de-livery of a variety of small therapeutic agents (Edeklev et al., 2019; Jain et al., 2019). Muna Aryal found that noninvasive transcranial low-intensity ultrasound can upregulate the glymphatic pathway, increase the whole-brain perivascular and parenchymal penetration of intrathecally administered small molecular agents, and increase the perivascular transport of larger agents, such as antibodies (Aryal et al., 2022). In this manner, this protocol can be used to directly bypass the blood-brain barrier for whole-brain delivery of a variety of agents. Continuous theta-burst stimulation (CTBS) is a crucial brain regulation technology. CTBS’s rapid regulation of cortical excitability and its relatively long-lasting aftereffects make it practical to regulate brain function (Di Lazzaro et al., 2005; Huang et al., 2011). CTBS could increase glymphatic fluid transport, especially CSF and ISF exchange, mediated by improved AQP4 polarization. In addition, the accelerated glymphatic pathway reduced Aβ deposition and enhanced spatial memory cognition, improving the pathological damage and clinical cognitive dysfunction of glymphatic dysfunction-related diseases (Wu et al., 2022). Moreover, CTBS significantly increased influx efficiency along the PVS and the efficiency of solute clearance, restored the loss of AQP4 polarization and improved anxiety-like behavior in sleep disorders animals (Liu et al., 2017). A spectrum of 40 Hz multimodal and noninvasive electrical or magnetic stimulation modalities can provide positive effects on brain function, disease pathology, and cognitive function in persons with AD (Blanco-Duque et al., 2023). The latest research show that multisensory gamma stimulation induces the influx of CSF and the efflux of ISF in the cortex of the AD mouse model, which was related with enhanced AQP4 polarization along astrocytic end feet and dilated mLVs (Murdock et al., 2024). So inhibiting glymphatic clearance prevented amyloid elimination via 40 Hz multisensory stimulation. Therefore, the study of the glymphatic system can provide new research ideas for central nervous system diseases, and may be a potential and promising new target for the treatment of central nervous system diseases.

## 7 Conclusion and perspectives

From the initial questioning of the glymphatic system, to the discovery of the glymphatic pathway, to the confirmation of mLVs and the continuous research progress, people have gradually clarified the circulation process and regulation of the glymphatic system. The glymphatic system maintains the stability of its internal environment via promoting the exchange of CSF and ISF through the perivascular spatial network system, transporting nutrients and neuroactive substances to the brain tissue, and eliminating metabolites from the brain tissue, and plays an important role in pathophysiological processes such as neurodegenerative diseases, stroke and brain injury. There are many factors that can affect the glymphatic system and mLVs during the perioperative period. Regulation of the glymphatic system and mLVs is anticipated to form a cluster intervention method during the perioperative period, and its associated research will contribute significantly to the advancement of clinical diagnosis and treatment of central nervous system diseases and the advancement of brain function research. Meanwhile, the glymphatic system provides new research perspectives for understanding the specific mechanisms of action of anesthetic drugs and general anesthesia states on the brain. Future translational medicine research should emphasize on improving perioperative brain protection measures from the perspective of enhancing the function of the glymphatic system to promote patient benefit.
